# Familial case of Muckle-Wells syndrome in the Russian population: a long way to the diagnosis

**DOI:** 10.1186/1546-0096-12-S1-P273

**Published:** 2014-09-17

**Authors:** Svetlana O Salugina, Evgeny Fedorov, Ekaterina Zaharova, Margarita Evsikova

**Affiliations:** 1 Research Centre of Medical Genetics (RCMG) of the Russian Academy of Medical Sciences (RAMS; 2 Children, Nasonova Research Institute of Rheumatology, Moscow, Russian Federation; 3 Nasonova Research Institute of Rheumatology, Moscow, Russian Federation

## Introduction

Muckle-Wells syndrome (MWS) is a rare auto-inflammatory disease related to the group of cryopyrin-associated periodic syndromes (CAPS) frequently complicated by amyloidosis. The disease is caused by mutation in NLRP3 (CIAS1) gene transmitted by autosomal dominant route.

## Objectives

To describe genetically verified familial MWS case in mother and daughter of the Russian ethnic origin.

## Methods

In addition to standard rheumatologist examination, NLRP3 gene was partially tested in the patient, her mother by direct partial sequencing.

## Results

### Case 1

female patient of 17 years old (daughter) has been examined in the Institute since the age of 8 (2004). Since the age of 10 months she has complained for periodic fever, transient macular and papular rash on different body areas without itching, arthralgia/arthritis without joint dysfunction, headache, abdominal pain, sore throat, nodal fever episodes, ulcerative stomatitis and genital ulcers. The patient has had a history of uveitis and frequent conjunctivitis since 8 years of age. Lucid asymptomatic intervals have been observed. Differential diagnosis: systemic juvenile arthritis, systemic vasculitis, periodic fever syndromes (FMF, TRAPS), Behcet’s disease since 2006.

### Case 2

female patient of 40 years old (mother) has been examined in the Institute since 31 years of age (2004). She has complained for periodic arthralgia/arthritis without joint dysfunction since 7 years. Since 17 the patient has developed fever, transient macular and papular rash on different body areas without itching, sore throat, nodal fever and ulcerative stomatitis episodes, genital and rectal ulcers, abdominal pains and frequent conjunctivitis. Sensorineural hearing loss with progressive deafness has developed since 2000 (27 years). Differential diagnosis: acute rheumatic fever, Still’s disease of adults, systemic lupus erythematosis, periodic fever syndromes (TRAPS), Behcet’s disease since 2006. Acute phase markers (ESR, CRP, leukocytosis) are permanently increased in both patients. HLAB51 is negative. Treatment with antibiotics, NSAID, disease-modifying anti-rheumatic drugs and colchicine had no effect and glucocorticoid therapy lead to temporary improvement. Molecular genetic test revealed NLRP3(CIAS1) - pThr350Met gene mutation in mother and daughter in heterozygous state. CAPS – MWS syndrome was diagnosed in 2013.

## Conclusion

MWS may occur in patients of the Russian ethnic origin and should be included in a range of differential conditions in patients with fever, rash and other inflammatory symptoms, particularly in the cases when similar signs are observed in the patient’s family or if standard anti-rheumatic and anti-inflammatory therapy has no adequate effect.

## Disclosure of interest

None declared.

